# Expression of Pluripotency Master Regulators during Two Key Developmental Transitions: EGA and Early Lineage Specification in the Bovine Embryo

**DOI:** 10.1371/journal.pone.0034110

**Published:** 2012-03-29

**Authors:** Daulat Raheem Khan, Delphine Dubé, Laurence Gall, Nathalie Peynot, Sylvie Ruffini, Ludivine Laffont, Daniel Le Bourhis, Séverine Degrelle, Alice Jouneau, Véronique Duranthon

**Affiliations:** 1 INRA UMR 1198 Biologie du Développement et de la Reproduction, Jouy en Josas, France; 2 ENVA, Maisons Alfort, France; 3 UNCEIA, Services Techniques, BP65, Maisons Alfort, France; University of Medicine and Dentistry of New Jersey, United States of America

## Abstract

Pluripotency genes are implicated in mouse embryonic genome activation (EGA) and pluripotent lineage specification. Moreover, their expression levels have been correlated with embryonic term development. In bovine, however, little information is available about dynamics of pluripotency genes during these processes. In this study, we charted quantitative and/or qualitative spatio-temporal expression patterns of transcripts and proteins of pluripotency genes (*OCT4, SOX2* and *NANOG*) and mRNA levels of some of their downstream targets in bovine oocytes and early embryos. Furthermore, to correlate expression patterns of these genes with term developmental potential, we used cloned embryos, having similar in vitro but different full term development rates. Our findings affirm: *firstly*, the core triad of pluripotency genes is probably not implicated in bovine EGA since their proteins were not detected during pre-EGA phase, despite the transcripts for *OCT4* and *SOX2* were present. *Secondly*, an earlier ICM specification of transcripts and proteins of *SOX2* and *NANOG* makes them pertinent candidates of bovine pluripotent lineage specification than *OCT4*. *Thirdly*, embryos with low term development potential have higher transcription rates; nevertheless, precarious balance between pluripotency genes is maintained. This balance presages normal in vitro development but, probably higher transcription rate disturbs it at later stage that abrogates term development.

## Introduction

Oogenesis is characterised by the accumulation of a myriad of maternal transcripts and proteins in the oocyte. These transcripts and proteins, referred to as maternal factors, are the products of “maternal-effect” genes. In the mouse, considerable information is available on maternal-effect genes and their roles in embryonic development. These factors endow oocytes with the ability to optimise follicular development, the maturation of germ cells, early embryonic development and particularly embryonic genome activation (EGA) [1] which is necessary to the transition from a maternal to an embryonic control of embryo development (MET).

In mice, embryonic genome activation is primarily elicited through an improved access of the transcription factors to the embryonic genome after its remodelling. These factors include a homeodomain containing transcription factor (*Oct4*/*Pou5f1*) and an SRY-related HMG-box containing factor (*Sox2*) [2]. Interestingly, *Oct4* and *Sox2* are expressed during oogenesis and their transcripts and proteins persist in the early embryo [3], [4]. The expression patterns of these factors presage their roles in embryonic development before and after EGA. Consistent with this idea, functional studies have demonstrated that before EGA, a depletion of *Oct4* at the 1-cell stage abrogates embryonic development [5] and critical levels of *Sox2* are necessary to achieve successful EGA [6]. After EGA *Sox2* deficient embryos halt their development at the morula stage and are unable to differentiate their trophectoderm [7]. At the blastocyst stage, *Oct4* is responsible for the lineage specification of the inner-cell-mass (ICM) and is down-regulated in the trophectoderm (TE) [3], whereas, SOX2 plays important role in maintaining the ICM through hetero-dimerization with OCT4, and its down-regulation leads to embryonic lethality [4].

It is worth mentioning here that *Oct4* and *Sox2* are not simply maternal-effect genes that are implicated in vivo; they also endow pluripotency on embryonic stem cells (ESC) in vitro [8]. Interestingly, the remarkable properties of ES cells are attributed to a set of three “master-regulators” :two of them are mentioned above (*Oct4* and *Sox2*) and the third actor is *Nanog* [9]. *Nanog* is not expressed in early embryos until morula and is not a maternal-effect gene [10]. In fact, *Nanog* is activated through the expression of *Oct4* and *Sox2* in mouse and human ES cells [11]. These three genes collaboratively control the “ground state” pluripotency in ES cells [12]. Furthermore, knockdown of any of these factors may prompt in vitro differentiation of ES cells. It has been observed that *Oct4* or *Sox2* knockdown direct ES cells to differentiate into the trophectoderm lineage [13], [14], [15], and *Nanog* ablation in human ES cells causes extra-embryonic endoderm lineage differentiation [16]. Moreover, the advent of direct reprogramming through the ectopic expression of pluripotency transcription factors has further emphasised their role. In fact, the retroviral induction of *Oct4* and *Sox2* in the human fibroblasts is sufficient to reprogram these cells to the pluripotent state (Induced pluripotent cells or iPS cells) [17] and they are also required for reprogramming mouse fibroblasts [18]. Understanding and dissecting the regulatory mechanisms that underlie the state of pluripotency have been the subject of wide ranging analyses. Indeed these three pluripotency factors elicit the transcriptional activation of other pluripotency genes through a positive feedback to themselves and the transcriptional repression of lineage-specifying factors [12].

In addition to early development and lineage specification, pluripotency genes have also been implicated in the long-term development potential of mouse embryos [19]. In this context, pluripotency transcription factors play pivotal roles in mice, since they control early embryonic development, EGA, pluripotent lineage (ICM) specification and ES cells derivation as well as long-term embryonic development. However, little information is available on these processes in bovine, despite the data that has accumulated to demonstrate species-specific differences in the processes of early development and lineage specification [20]. For example, the major burst of embryonic genome activation takes place after several cleavages (8–16 cell stage) in bovine, compared to the 2-cell stage in mice [21], with regulatory mechanisms that remain largely cryptic. Similarly, the establishment of the pluripotent lineage proceeds differently in different species. This is evident from the fact that *Oct4* is expressed in the ICM and TE of bovine and porcine blastocysts [20], [22], [23]. Moreover, to our knowledge, the spatio-temporal expression profile of *Sox2* has not yet been addressed in bovine embryos. However, NANOG protein is found to be ICM specific in bovine blastocysts [24]. It is therefore speculated that due to the lack of information on these basic processes and their controlling mechanisms in bovine embryos, efforts to derive bovine ES cells have largely been fruitless [25].

In addition to the species-specific differences, defective development and female sterility can hamper the development of gene-targeting experiments to study the process of bovine MET. However, somatic cell nuclear reprogramming in cloning experiments represents an interesting tool to study the important events during development in bovine. In nuclear transplantation (cloning) a differentiated somatic cell nucleus is transformed into an undifferentiated totipotent (capable of developing into a whole individual) state when inserted in an enucleated oocyte. This implies huge epigenetic changes that result in a transition from a somatic to an embryonic gene expression pattern and is referred to as “nuclear reprogramming” [26]. And indeed, nuclear reprogramming in cloning experiments is equivalent to MET in a context of natural fertilization insofar as both result in establishment of totipotency [27]. In fact, nuclear reprogramming results from nucleo-ooplasmic interactions. Interestingly, the effects of different cell types in cloning efficiency have already been documented [28]; moreover, a variation in full-term development potential has been observed using different cell lines of the same cell type [27], [29], which could be anticipated by means of gene expression analyses as early as the morula stage in bovine [30]. We therefore speculated that identifying aberrations in the pluripotency gene expression patterns of cloned morulae derived from somatic cell nuclear transfer (SCNT) from cell lines with different term developmental potentials might be an interesting approach to study the role of these genes in optimum MET and long-term embryonic development.

In order to address this issue, the objectives of the present study in bovine were *firstly*, to reveal the dynamics of the pluripotency genes products (mRNA and proteins) during two key developmental steps: EGA, and establishment of the pluripotent lineage during blastocyst formation and *secondly*, to consider a possible relationship between the levels of pluripotency genes expression and the long-term development potential of bovine embryos using cloned bovine embryos.

## Materials and Methods

### 1. Preparation and Collection of Biological Material

Gene expression analyses of genes of pluripotency and their downstream targets were performed on the same batches of embryos/oocytes.

#### 1.1 Immature, Mature Oocytes and IVF Embryos

Bovine ovaries were collected from the slaughterhouse (Socopa, cours Saint Paul, 27110 Le Neubourg, France); oocytes with diameter of 2–7 mm were aspirated from the follicles. Some of the immature oocytes (GV) used for gene expression analysis were dry-frozen after the selection and removal of cumulus cells by gentle pipetting. The rest of the selected cumulus oocyte complexes (COCs) were placed in the maturation medium [31]. Mature oocytes and fertilized embryos were obtained after in vitro oocyte maturation, fertilization and embryo culture as described previously [32], [33]. Mature oocytes (MII) intended to be used for gene expression analysis were collected 24 hrs post maturation (hpm) and cumulus cells were removed after treatment with hyaluronidase (HEPES-buffered TCM 199 with 0.5% hyaluronidase). The denuded MII oocytes were then dry-frozen. IVF embryos were also collected and frozen at appropriate stages. Generally, 4-cell embryos (4-cell) were recovered 41 hrs post-insemination (hpi), 8 to 16-cell embryos at 72 hpi, early morulae were collected at 120 hpi and blastocysts were obtained at day 7 (E7) post-insemination. Furthermore, the embryos for in situ hybridization and immunofluorescence were also collected at days 7 to 9 (E7, E8 and E9) post-insemination.

#### 1.2 Somatic Donor Cell Culture

Primary cultures of bovine fibroblasts were derived from ear skin biopsies of two separate Holstein heifers, OV5538 (here named Somatic cell A) and OV029 (here named Somatic cell B). These cells were frozen-stored at passage 1 (previously described in [30], [34]) and used as sources of donor nuclei for Nuclear Transfer (NT) between passages 3 to 12. Donor fibroblasts were grown for 5 days in Dulbecco's Modified Eagle Medium (DMEM; Life Technologies, Cergy, France) supplemented with 10% FCS (Life Technologies) at 38°C with 5% CO2. During this period the cells reached confluence and were synchronized to G0/G1 of the cell cycle through contact inhibition. Nuclear donor cells were trypsinized at 37°C for 5 min and were re-suspended in 1 mL DMEM supplemented with 10% fetal calf serum (FCS) for nuclear transfer. Somatic cells for gene expression analysis were also cultured as described above.

#### 1.3 Nuclear Transfer Embryos

Recipient oocytes were matured in vitro as previously described [32] and enucleated at 20–22 hpm (hours post-maturation). SCNT embryos were reconstructed by the electrofusion of enucleated oocytes with donor cells at 23–24 hpm (2.0 kV/cm 30 µs ×2 pulses). The reconstructed embryos were activated by incubation for 5 h after fusion, in 10 µg/ml cycloheximide (Sigma) and 5 µg/ml cytochalasin B (Sigma) in TCM 199 Medium with 10% fetal calf serum. Activated embryos were co-cultured under the same conditions as the IVF embryos [33]. Grade 1 morulae, defined as in [35] (Clone Morula A and Clone Morula B) were selected at 120 hours post-fusion and immediately dry-frozen for further molecular analysis.

### 2. Spatio-temporal Gene Expression Analysis

#### 2.1 RNA Extraction and Reverse Transcription

Embryonic total RNAs were extracted from batches of embryos (n = 30 embryos) using the PicoPure RNA extraction kit (Arcturus). The challenges of a loss of embryonic RNA during column purification and later the normalization of qRT-PCR data were addressed through the addition of a carrier RNA (16S–28S carrier, Roche Diagnostics, Meylan, France) and an exogenous transcript (Luciferase, Promega) at the time of extraction. In order to enhance the RNA recovery rate and estimate the number of ‘equivalent embryos’ in the sample after purification, we added 2.5 µg carrier RNA to the samples. The carrier RNA recovery rate was estimated by OD measurement, while embryonic RNA was considered to be negligible when compared to the carrier RNA. This recovery rate was taken into account to calculate the number of equivalent embryos left in each sample after column purification. In addition, luciferase encoding reporter transcripts was added 1 pg per embryo along with carrier for qRT-PCR data normalisation as an exogenous control. A purification procedure using DNAse I (Qiagen) treatment at 25°C for 15 min was performed prior to elution.

Total RNA was subjected to RT-PCR and the cDNAs were synthesized using the Superscript III enzyme (Invitrogen) and hexamer random primers (Roche Diagnostics, Meylan, France) in 20 µl final volume. RT-PCR products were diluted so as to obtain 1 equivalent embryo per 10 µl. Quantitative PCR was performed on 0.5 or 1 equivalent embryos per well in 96-well plates, depending upon the gene of interest.

The RNA extraction of somatic cells was performed in order to obtain three independent biological quantifications. Four dishes were cultured concomitantly to confluence from a single cell suspension. One dish was used to count the cells, while cells from the three other dishes were recovered for RNA extraction using the Qiagen minikit. Total RNAs were subjected to cDNA synthesis as described for embryos. The cDNAs of somatic cells were diluted to obtain an equivalent of 3000 cells in 10 µl of RT product.

#### 2.2 Real Time qRT-PCR

Before performing qRT-PCR on the samples included in these experiments, the specificity of the primers was validated for the IVF embryos and further verified on 2% agarose gel. Primer sequences are provided in the table 1. Moreover, the appropriate number of equivalent embryos required per well for each gene was determined by analysing the correlation between the number of copies obtained for 0.5, 1 and 2 equivalent embryos per well using the same concentration of primers. The reactions were performed on an ABI Prism 7000 sequence detector (Applied Biosystems). Each gene was run separately and a 10-fold dilution series of quantified amplicon was included in each run to determine the standard curve. This experiment consisted of three biological repetitions and three technical replicates for each PCR reaction (n = 9 for each sample stage) as well as for the standard curve. The cDNA consisting of an appropriate number of equivalent embryos or somatic cells in a volume of 10 µl was added to the PCR mix containing 12.5 µl of 2× SybrGreen Mastermix (Applied Biosystems, Courtaboeuf, France), 0.25 µl Uracyl N-glycosylase (1 U/µl), 0.5 µl of each primer (10 µM initial concentration) and 1.25 µl H_2_O to obtain a final volume of 25 µl. The thermal cyclic profile started with a 2 min step at 50°C, followed by 10 min at 95°C, and 45 cycles each consisting of denaturation for 15 sec at 95°C, and annealing and extension for 60 sec at 60°C. Dissociation curves were obtained after each PCR run to ensure that a single PCR product had been amplified.

**Table 1:** Primers used for the qRT-PCR of different genes.

Name	Primer Sequence	Product Size
OCT4	F:GGCGCCAGAGGAAAGGATACR:AGAAGGGCAAACGATCAAGCA	173
SOX2	F:CCATGCAGGTTGACATCGTR:ACACAACTACGGAAACTAAAAGTGG	184
NANOG	F:AACAACTGGCCGAGGAATAGR:AGGAGTGGTTGCTCCAAGAC	193
STAT3	F:GACCTTTTCAGATAAGAGGGAGACAR:GCAGCAGGAAATCTCCAAGGA	198
HESX1	F:ACTGTGTTCCATCCACGAAACCR:CAAACACTTTCTTCCGGGACTG	152
MEIS1	F:TCGGGAAGGATGGGAAAR:CCAAGGTGGGACTATGGAAA	289
NODAL	F:CTCCGCTTCCCATAGCAGR:CCTGTTCACTGTCACTCTGTCC	206
ISL1	F:TTATCATTGGGCTGCTGTTGR:CCTGCTATGCCGCTAACC	169
SDHA	F: GCAGAACCTGATGCTTTGTGR: CGTAGGAGAGCGTGTGCTT	185
YWHAZ	F: GCATCCCACAGACTATTTCCR: GCAAAGACAATGACAGACCAs	120
GAPD	F: TTCAACGGCACAGTCAAGGR: ACATACTCAGCACCAGCATCAC	119

The numbers of copies of each gene were determined based upon their standard curves. The qRT-PCR data were normalised using the geometric mean of the endogenous genes GAPD, SDHA and YWHAZ, which have been shown to be an appropriate set of reference genes for pre-implantation bovine embryos [36], while exogenous normalisation with luciferase was performed in the case of cloned embryos [30]. Three biological repetitions were performed for each developmental stage and the mean Ct value for each repetition was obtained from a technical triplicate. The number of copies for each biological repetition was calculated using a standard dilution curve obtained with each reaction. The ratio values for each stage of embryos were obtained from each biological repetition divided by the geometric mean of the control genes corresponding to that biological repetition, and then the mean of the three biological repetitions was taken. Error bars represent ±SEM.

#### 2.3 In situ Hybridization (ISH)

The bovine cDNA fragments encoding *OCT4*, *NANOG* and *SOX2* were derived from PCR amplifications, sequenced, cloned into pGEMT-Easy (Promega) and in vitro transcribed using the Sp6 RNA polymerase for anti-sense probes and T7 RNA polymerase to generate the sense probes as described in [37]. The *OCT4*, *NANOG* and *SOX2* antisens probes correspond to the NCBI entries DQ126156, DQ126153.1 and DQ126150 respectively. The blastocysts at E7, E8 and E9 were fixed in 4% paraformaldehyde and subjected to whole-mount in situ hybridization as described in [38], [39]. Briefly, samples were permeabilised with proteinase K (10 µg/ml in PBS-Tween) for 10 mn before hybridization with Dig-labelled riboprobes. After incubation with anti-Dig antibody (Roche), samples were incubated in BM purple (Roche) until the colour developed. The hybridized embryos were observed under an inverted microscope and photographed using a digital camera (Zeiss).

### 3. Spatio-temporal Protein Expression Pattern

#### 3.1 Immunofluorescence

Immature (GV), mature bovine oocytes (MII), 4-cell, 8-cell, 16-cell, early morula (25–30 cells) and blastocysts at days E7, E8 and E9 were fixed in 4% (w/v) paraformaldehyde (PAF). The oocytes and embryos were then washed in PBS for 10 min. The samples were permeabilized with 1.0% (v/v) Triton X-100/PBS for 1 hr at room temperature. The zona pellucida of embryos at the blastocyst stage was removed and the embryos were opened manually to enable better access for the antibodies to the ICM. Furthermore, oocytes and embryos were boiled in 10 mM sodium citrate buffer [40] and then maintained at a sub-boiling temperature for 10 min for the antigen unmasking procedure. The samples were rinsed in PBS and then incubated with 2% (w/v) BSA-PBS for 1 h. Then the oocytes and embryos were incubated with primary antibody diluted in 2% (w/v) BSA-PBS for 2 hr at room temperature (RT). The embryos were then rinsed in PBS solution and incubated with secondary antibody diluted in PBS-BSA for 45 min at RT. After washing with PBS, the nuclei of the embryos were counter-stained with DAPI and mounted on the glass slides with anti-fading medium (Vectashield, Vector Laboratories, Burlingame, CA, USA). The immunofluorescent–labelled oocytes and embryos were observed under a fluorescent microscope (Axioplan imaging Apotome apparatus, Zeiss, Germany) (MIMA2 Platform, INRA, Jouy en Josas, France).

#### 3.2 Western Blot

Immature (GV), mature bovine oocytes (MII), 4-cell, 8-cell, 16-cell, early morula (25–30 cells) and blastocysts at days E7, E8 and E9 were lysed with sodium dodecyl sulfate (SDS)-buffer. The embryonic polypeptides were separated through 4–12% Bis- Tris Gel NuPage gel electrophoresis, as previously described [41]. Protein molecular weight markers (14–200 kD, Amersham) were run simultaneously as molecular weight standards. Electrophoretically separated polypeptides were transferred onto a polyvinylidene difluoride membrane (Hybond-P PVDF, Amersham). The membranes were blocked with 1/1000 Tween 20-PBS (PBS-T, Prolabo, France) containing 4% (w/v) non-fat dried milk. The membranes were then incubated with primary antibodies for 2 hrs at room temperature and washed three times with PBS-T and incubated with secondary anti-rabbit antibody conjugated with peroxidase, followed again by washing three times in PBS-T. Peroxidase activity was revealed using the ECL-Plus Western Blot detection system (Amersham). The signals were analysed using the Image Analysis system (Advanced Image Data Analyzer software, LAS 1000 camera, Fuji films).

#### Primary antibodies

OCT4 protein was labelled with a rabbit polyclonal anti-OCT4 antibody (Abcam; 1∶150 in PBS–BSA for immunofluorescence and 1/500 in PBS –T 4% non-fat dried milk for Western blot). NANOG was labelled with a rabbit polyclonal anti-NANOG antibody (Reprotech; 1∶500 in PBS–BSA for immunofluorescence and 1/1000 in PBS–T 4% non-fat dried milk for Western blot). SOX2 was labelled with a mouse monoclonal anti SOX2 antibody (R&D Systems; 1∶50 in PBS–BSA for immunofluorescence and 1/250 in PBS –T4% non-fat dried milk for Western blot).

#### Secondary antibodies

A fluorescein isothiocyanate (FITC)-labelled anti-rabbit secondary antibody (Jackson Immunoresearch; 1∶500) or a peroxydase-conjugated anti-rabbit IgG antibody (1/5000; Santa Cruz Biotechnology), were used.

### Statistical Analysis

The statistical significance of the levels of gene expression patterns was determined using one-way ANOVA with STATISTICA 9.1 software (StatSoft, Inc. USA.).

## Results

### 1. Expression of *OCT4*, *SOX2* and *NANOG* in early bovine embryos

First of all, we charted the temporal expression profiles of the three principal pluripotency genes (*OCT4*, *SOX2* and *NANOG*) in oocytes and early bovine embryos using qRT-PCR. The quantification results were normalized using the geometric mean of the three endogenous genes *GAPD, SDHA* and *YWHAZ*, because this has been shown to be the best combination of housekeeping genes for the normalization of qPCR data during early development in bovine [36]. Under these conditions, the qRT-PCR results revealed variations in the relative expressions of candidate genes in the total transcriptome of the embryo. In addition, we articulated the spatial compartmentalization of these transcripts into the earliest lineages (ICM and TE) from a qualitative point of view, using in situ hybridization in bovine blastocysts. Immunofluorescence was then performed on OCT4, SOX2 and NANOG to detect the proteins, and the results were further confirmed by Western Blot analysis.

**Figure 1:**
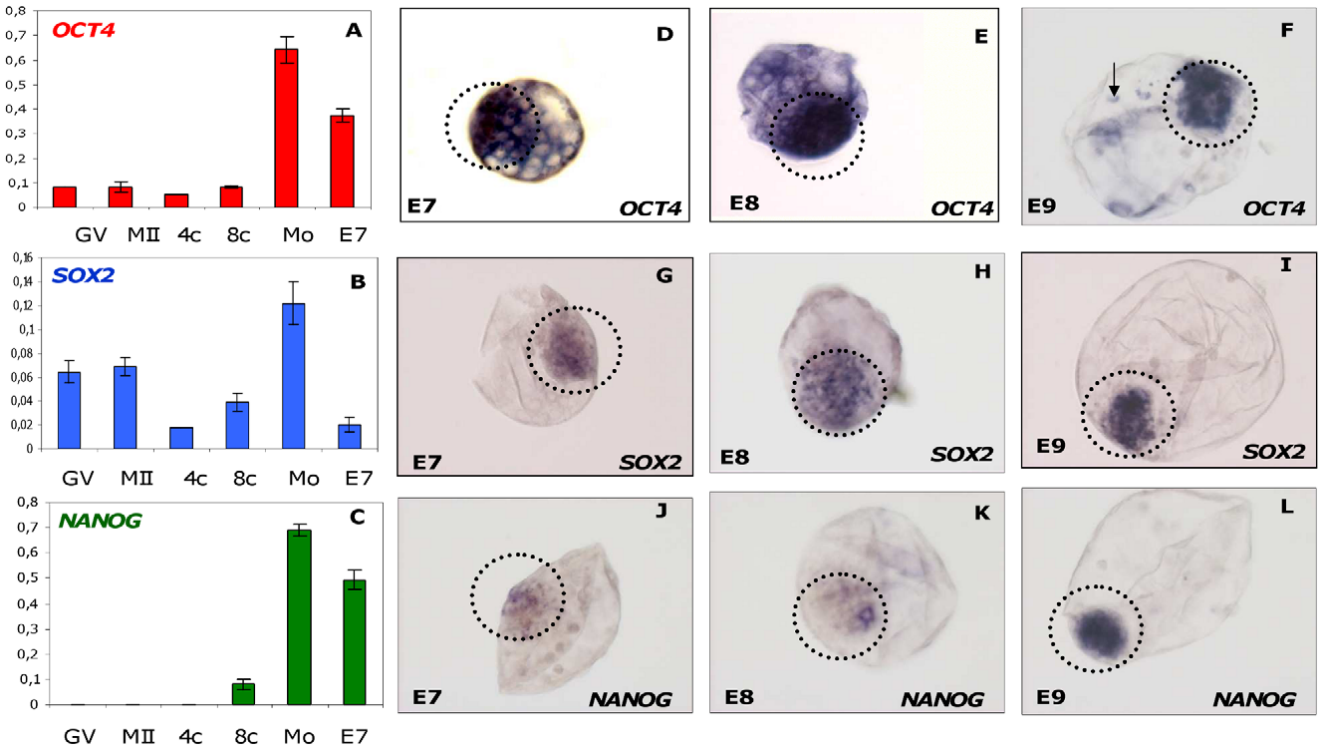
Expression profiles and transcript localization of pluripotency genes during bovine pre-implantation development of the bovine embryos. Expression profiles of *OCT4*, *SOX2* and *NANOG* (**A–C**) were charted using qRT-PCR at pre-implantation stages of bovine oocytes/embryos: GV (Germinal Vesicle), MII (Metaphase 2 oocyte), 4-cell stage, 8–16-cell stage, Morula (25–30 cells) and E7 (Day 7 blastocyst). The results were normalised using the geometric mean of the endogenous *GAPD, SDHA* and *YWHAZ* genes. Values are means ± SEM of one equivalent embryo/oocyte. The experiment was repeated three times and each repetition contained a triplicate of each sample stage. The localization and compartmentalization of *OCT4* (D–F), *SOX2* (G–I) and *NANOG* (J–L) was determined by whole mount in situ hybridization (ISH) in a spatio-temporal manner from day7 to day9 (E7, E8 and E9) blastocysts. Encircled regions demarcate the ICM.

*OCT4* transcripts were detected in the oocytes at the germinal vesicles (GV) stage, although at low levels. The levels of *OCT4* mRNA remained unchanged until the 8–16 cell stage. The highest level of *OCT4* expression was observed at the early morula stage (25–30 cells). Likewise, *OCT4* expression remained high at the blastocyst stage (E7); however, its relative level was significantly lower than in the morula (Fig. 1A). In situ hybridization revealed the ubiquitous presence of *OCT4* in the ICM and trophectoderm at E7 and E8 (Fig. 1D–E). By contrast, at E9, *OCT4* was restricted to the ICM although a few cells were still positive in the TE (Fig. 1F).

**Figure 2:**
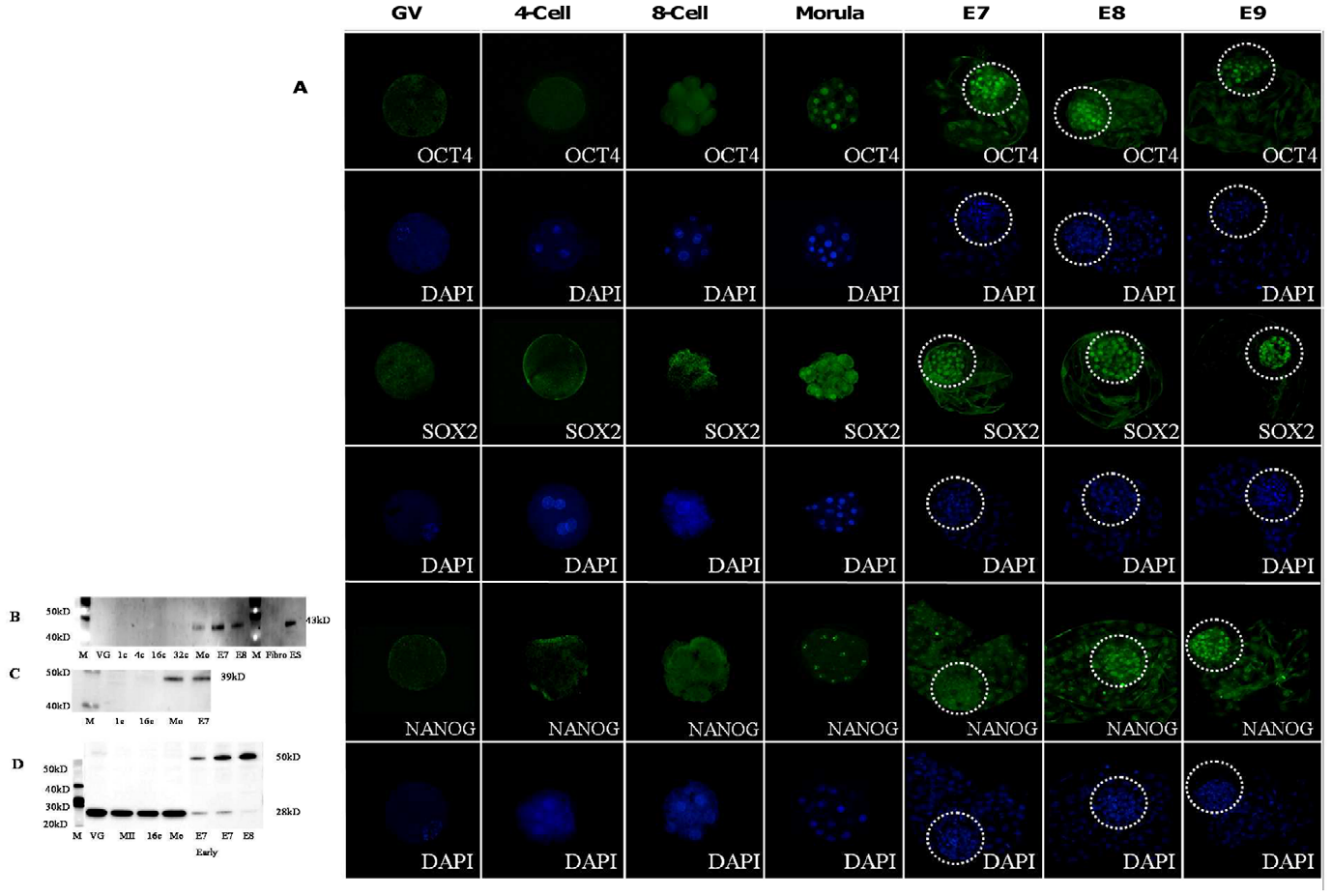
Protein expression of pluripotency genes in the early bovine embryos. Immunofluorescent detection of OCT4, SOX2 and NANOG protein was done in the pre-implantation bovine embryos/oocytes: GV (Germinal Vesicle), MII (Metaphase 2 oocyte), 4-cell stage, 8–16-cell stage and Morula (25–30 cells) using immunofluorescence (A) and Western Blot (B-D). Protein compartmentalization in the ICM/TE was also studied at E7 (Day 7 blastocyst), E8 (Day 8 blastocyst) and E9 (Day 9 blastocyst) as well. Encircled areas demarcate the ICM.

Using immunofluorescence, OCT4 protein was not detectable in oocytes and early embryos until the 8-cell stage. At the 16-cell stage (which marks the end of EGA), a very weak OCT4 signal was detected in blastomere nuclei (data not shown). The early morula stage was the first point at which we detected a strong OCT4 labelling in all nuclei. OCT4 protein in the E7, E8 and E9 blastocysts was detectable in cells of both the ICM and TE, but, the nuclei of ICM blastomeres displayed apparently stronger labelling than the TE at all these stages. Moreover, we observed a gradual tendency towards OCT4 protein restriction in the ICM from E7 to E8 and E9 (Fig. 2A). Immunofluorescence detection findings agreed with the Western Blot analysis which revealed a polypeptide at 43 kD in the morula (more than 32 cells) and blastocyst (Fig. 2B). The earlier stages were negative for OCT4. Furthermore, we confirmed the antibody specificity by using mouse ES cells as positive controls and bovine fibroblast cells as negative controls (Fig. 2B).

In bovine, *SOX2* mRNA was detected in the GV and metaphase II (MII) oocyte stages. The relative level of *SOX2* fell significantly at the 4-cell stage but tended to increase at the 8–16 cell stage, leading to the highest level of *SOX2* in the early morula transcriptome. Nevertheless, a major decrease in the relative level of expression was observed at the E7 blastocyst stage (Fig. 1B). *In situ* hybridization experiments revealed a restriction of *SOX2* transcripts to the ICM at E8 and E9. Trophectoderm cells, however, were weakly labelled in their cytoplasm at E7. This labelling disappeared at E8 and E9, while the ICM became more intensely labelled (Fig. 1G–I).

The specific labelling of SOX2 protein was not detected until the 8-cell stage using immunofluorescence. The earliest specific detection of SOX2 was in the nuclei of early morulae. Interestingly, SOX2 protein was detected in E7, E8 and E9 blastocysts. At these stages only the nuclei of ICM blastomeres were labelled and the TE was negative (Fig. 2A). Furthermore, our results were confirmed using Western Blot, which showed a single polypeptide of 39 kD (the expected molecular weight) that was only detectable in the morula and blastocyst (Fig. 2C).

The *NANOG* transcript was not detectable using qRT-PCR in the GV, MII oocytes or 4 cell stage embryos. *NANOG* transcript levels started to rise above background level at the 8–16-cell stage. *NANOG* was at its peak at the early morula stage and then slightly but non-significantly declined at E7 blastocyst (Fig. 1C). *In situ* hybridization was sufficiently sensitive to detect *NANOG* in the ICM of E7, E8 and E9 blastocysts compared to the negative controls, while TE was not labelled at all (Fig. 1J–L).

We did not observe any specific nuclear labelling of the NANOG protein between the oocyte and the 8-cell stage (Fig. 2A) and even 16-cell stage (data not shown), using immunofluorescence. At the early morula stage, a specific nucleolar labelling was seen in all blastomeres which persisted until E9. In addition of this, in the ICM a specific nucleoplasmic labelling persisted from E7 to E9. Western Blot analysis revealed the existence of two NANOG protein polypeptides in early bovine embryos, at 28 kD from the oocyte to the morula stage and at 50 kD at the blastocyst stage (Fig. 2D). We assumed the possibility of NANOG dimerization, in accordance with data in the literature [42], which had reported that NANOG forms homodimers, detectable by Western Blot in order to exert its pluripotency function.

### 2. Expression profile of few genes involved in Signalling Pathway or Early Patterning in early bovine embryos

**Figure 3:**
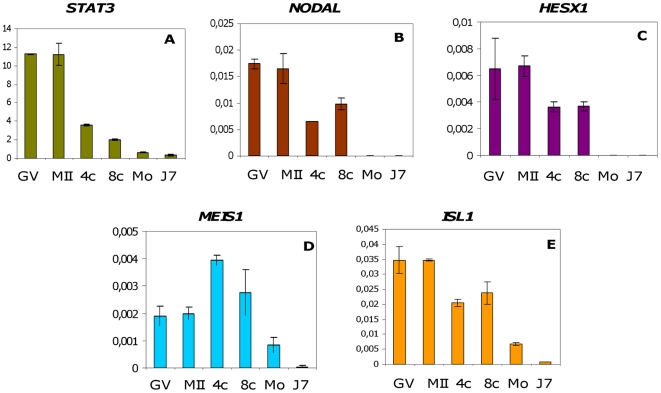
Expression patterns of the ST/EP genes. Expression profiles of *STAT3, NODAL, HESX1, MEIS1* and *ISL1* (**A–E**) were charted using qRT-PCR during the pre-implantation stages of bovine oocytes/embryos: GV (Germinal Vesicle), MII (Metaphase 2 oocyte), 4-cell stage, 8–16-cell stage, Morula (25–30 cells) and E7 (Day 7 blastocyst). The results were normalised using the geometric mean of endogenous *GAPD, SDHA* and *YWHAZ* genes. Values are means ± SEM of one equivalent embryo/oocyte. The experiment was repeated three times and each repetition contained a triplicate of each sample stage.

To further investigate pluripotency reprogramming in early bovine embryos, we analyzed the expression patterns of few genes known to be involved either in signalling pathways regulating pluripotency or in early patterning in the mouse model. Two genes involved two different signalling pathways in ES cells were chosen: *Stat3* belongs to a JAK-STAT signalling pathway, while *Nodal* is a member of the TGF-beta family that constitutes an alternate signalling pathway. We choose *HESX1, MEIS 1* and *ISL1* as early patterning genes because they encode transcription factors involved in numerous functions during embryo development and because master regulators of pluripotency *OCT4, SOX2* and *NANOG*) have been shown to bind their regulatory regions in ES cells [12]. We therefore analysed the expression patterns of these five genes (hereafter referred to as SP/EP for Signalling Pathway/Early Patterning genes) in bovine embryos using qRT-PCR (Fig. 3A–E). The expression profiles of these genes revealed their overall resemblance during the course of pre-implantation development, except for *MEIS1*. In fact, the transcripts of these genes were abundant in GV and MII oocytes, whereas after fertilization they gradually regressed to reach their lowest concentrations at the blastocyst stage. However, *MEIS1* increased transiently at the 4-cell stage and then declined progressively until the blastocyst stage.

### 3. Expression patterns of pluripotency genes and SP/EP genes in cloned bovine embryos

In this part of the study, we investigated the reprogramming of pluripotency genes in cloned bovine early morulae with different potentials (A = 12.7% vs. B = 1.8%, referred to respectively as 5538 and 029 in [30] for full-term development). This difference depends upon the origin of the fibroblasts used as nuclear donors, which are derived from two different animals. Here, we studied the expression levels of the pluripotency genes in bovine morulae derived through cloning and compared them with control IVF morulae as well as with their donor somatic cells.

**Figure 4:**
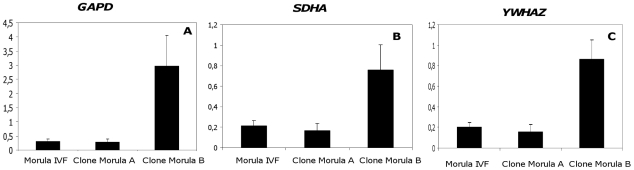
Expression patterns of endogenous control genes in bovine IVF and cloned embryos. Expression levels of the endogenous control genes *GAPD, SDHA* and *YWHAZ* were determined in bovine IVF and cloned morulae (Clone Morula A = better developmental potential and Clone Morula B = poorer developmental potential). Results were normalised with exogenous transcript luciferase.

It is worth mentioning here that the global transcriptome of cloned embryos is not directly comparable to that of fertilized embryos [27], [30]. Therefore, the genes used to normalise qRT-PCR results in the fertilized embryos could not be used in the cloned embryos unless otherwise tested. Since there have so far been no studies that have tested endogenous genes for the normalisation of qRT-PCR results comparing cloned and IVF bovine embryos, we adopted a two step strategy. *Firstly*, we supposed that if the expression levels of the three endogenous control genes *GAPD, SDHA* and *YWHAZ* in the three types of morulae (two types of cloned and IVF morulae) were similar, these genes could be used as endogenous reference genes. However, our findings showed that the expression pattern of each of these endogenous control genes was not uniform between the three types of morulae when normalised to an exogenous luciferase transcript added at a constant level per embryo in each sample at the time of RNA extraction. B cloned morulae expressed higher levels of these genes on a per embryo basis (Fig. 4A–C). *Secondly*, because the expression levels of the endogenous genes were not similar; these genes could not be used as qRT-PCR control genes. We therefore decided to use the exogenous luciferase transcript, added at a constant level per embryo, as a reporter gene to normalise the results of qRT-PCR when comparing cloned and IVF early morulae.

**Figure 5:**
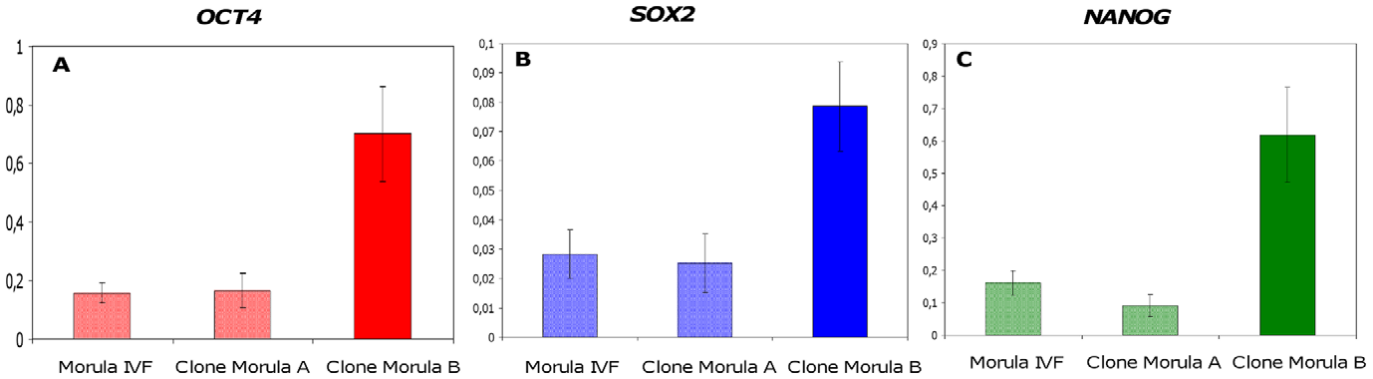
Expression patterns of genes of pluripotency in bovine cloned embryos. Expression patterns of *OCT4*, *SOX2* and *NANOG* were determined in bovine cloned morulae (Clone Morula A and Clone Morula B) and were compared with the levels of expression of these genes in controls (IVF Morulae). The results of qRT-PCR in all three types of morulae were normalised using an exogenous transcript luciferase. Values are means ± SEM of one equivalent embryo. The experiment was repeated three times and each repetition contained a triplicate of each sample.

**Figure 6:**
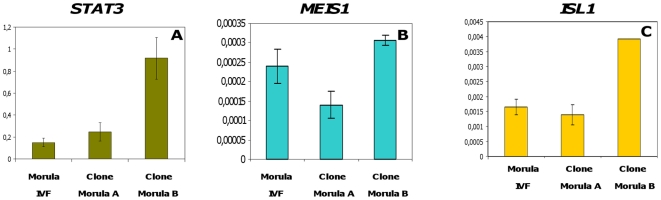
Expression patterns of SP/EP genes in bovine cloned embryos. Expression levels of *STAT3, NODAL, HESX1, MEIS1* and *ISL1* were determined in bovine cloned morulae (Clone Morula A = better developmental potential and Clone Morula B = poorer developmental potential) and were compared with the levels of these genes in control IVF morulae (Morula). *NODAL* and *HESX1* were not detectable at morula stage so the data is not shown. The results of qRT-PCR were normalised using an exogenous transcript luciferase. Values are means ± SEM of one equivalent embryo/oocyte. The experiment was repeated three times and each repetition contained a triplicate of each sample.

Under these conditions, we observed no differences in the levels of expression of any of the three pluripotency genes between the fertilised embryo and the cloned embryo with better term developmental potential (Clone Morula A). However, Clone Embryo B (with poor developmental potential until term) displayed significantly higher levels of expression of all three pluripotency genes (Fig. 5A–C). Furthermore, we compared the expression levels of the SP/EP genes with quantifiable expression at the morula stage; i.e. *STAT3*, *ISL1* and *MEIS1*, in both cloned and IVF morulae and observed that *STAT3* and *ISL1* were expressed at significantly higher level in clone Morula B than in Clone Morula A and the IVF controls. However, there was no significant difference in *MEIS1* expression between the three types of morulae (Fig. 6A–C).

**Figure 7:**
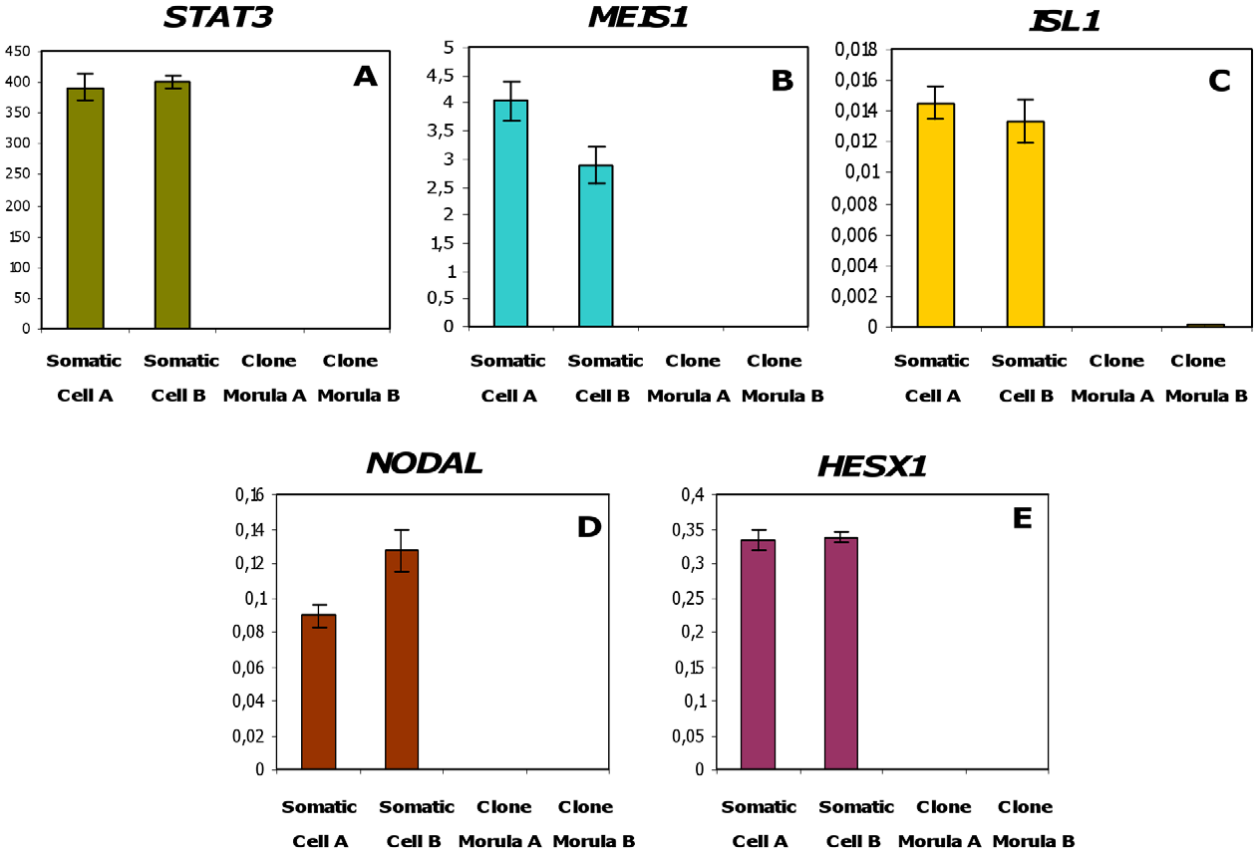
Expression patterns of SP/EP genes in the somatic donor cells and bovine cloned embryos derived from these cells. Expression levels of *STAT3, NODAL, HESX1, MEIS1* and *ISL1* were determined in bovine cloned morulae (Clone Morula A = better developmental potential and Clone Morula B = poorer developmental potential) and their somatic donor cells (Somatic Cell A = Fibroblast cells 5538 and Somatic Cell B = Fibroblast cells 029). The data represents number of transcript molecules in a single cell in the somatic cells as well as cloned morulae. The results of qRT-PCR were normalised using an exogenous transcript luciferase. Values are means ± SEM of one equivalent cell. The experiment was repeated three times and each repetition contained a triplicate of each sample.

We therefore considered whether the higher expression of pluripotency genes and SP/EP genes in cloned B morulae could be due to a differential expression of these genes in donor cells. We thus compared the expression levels of the pluripotency genes and SP/EP genes in the two types of somatic donor cells. However, we were not able to quantify pluripotency gene expression in donor cells: even with 3000 cells, the Ct for qRT-PCR experiments were out of the reference scale, despite the high sensitivity of our PCR conditions that made it possible to quantify about ten transcripts per sample (data not shown). We thus concluded that these genes were not expressed in donor fibroblasts, whatever their origin (A or B). Performing the same comparison with SP/EP genes, we were unable to evidence any difference in expression between A and B donor cells (Figure 7 A–E). These genes were significantly down-regulated after nuclear reprogramming, as illustrated by a per cell comparison of their expression in donor fibroblasts and in cloned embryos. We thus concluded that the higher expression of the pluripotency genes and most of the SP/EP genes in clone morulae with poorer full-term development potential was not due to their previously higher expression in the corresponding donor cells.

**Figure 8:**
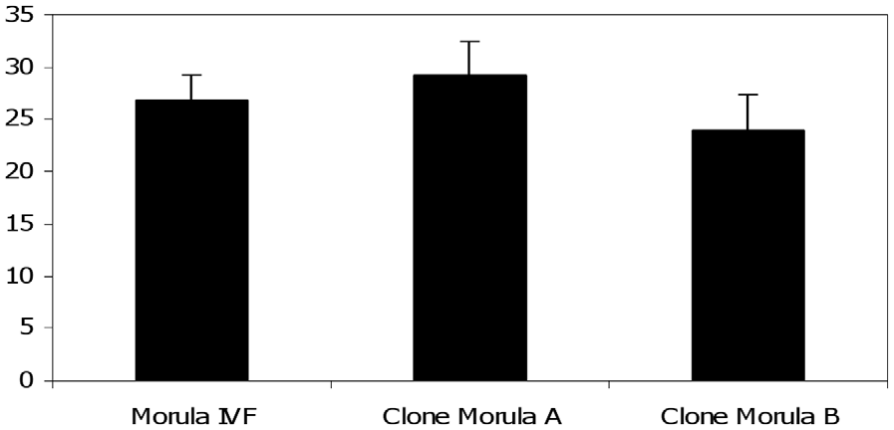
Number of cells in early bovine IVF and cloned morulae. The number of cells in each type of morula (Morula (IVF), Clone Morula A and Clone Morula B) was counted by staining the nuclei. Thirty embryos were used to calculate the number of cells derived from three different trials.

The observation concerning higher levels of pluripotency and SP/EP transcripts in poor quality clones presented an interesting paradox when trying to derive any conclusions from these results. We supposed that because normalisation with an exogenous transcript provided a per embryo-based quantification of a specific transcript rather than its relative abundance within an embryo, the observation of higher levels of pluripotency and SP/EP transcripts in these poor quality clones might either be due to a larger number of cells in these embryos or a generally larger quantity of all transcripts leading to a higher mRNA content. In order to address this problem despite the morphological similarity of the three types of early morulae we analysed, it was decided to precisely count the number of cells in all three types of morulae (two types of clones and the fertilized morulae). No significant difference was seen in the number of cells contained in the three types of morulae (Fig. 8). It was therefore possible to conclude as to the existence of differences in the expression levels of numerous genes, including the pluripotency genes, between cloned embryos with poorer potentials for full-term development and fertilized embryos.

## Discussion

To our knowledge, this is the first study in bovine to have simultaneously revealed spatio-temporal expression patterns of the transcripts and proteins of the core pluripotency genes (*OCT4, SOX2* and *NANOG*) and the mRNA expression levels of the five SP/EP genes in oocytes and early embryos.

### 1. Expression of *OCT4*, *SOX2* and *NANOG* in bovine early embryos

#### a) Expression profiles and spatial distribution of pluripotency transcripts

Our findings showed maternal contribution of the *OCT4* and *SOX2* mRNA in bovine embryos. Furthermore, the highest levels of all three pluripotency genes at morula stage indicated that these genes are also transcribed by the embryo after EGA. These observations agree with the findings of studies in mice [2], [43]. In bovine, similar *OCT4* expression has been documented in qualitative and semi-quantitative studies [22], [44], but the present study reports for the first time on *SOX2* and *NANOG* expression in multiple early stages of bovine embryos.

The qualitative spatial distribution analysis of *OCT4* and *SOX2* transcripts revealed their ubiquitous presence in the ICM and TE at E7, while the *NANOG* transcripts were found to be ICM-specific as soon as the blastocyst formed. The presence of *OCT4* in the ICM and TE corroborates previous findings in bovine [23], while in mice, *OCT4* and *SOX2* transcripts are ICM-specific as soon as the formation of the blastocyst [3], [4]. *NANOG* specificity in the ICM in bovine, on the other hand, was similar to that seen in mice and humans [43]. We consider this finding to be significant with respect to early lineage differentiation in bovine. Indeed, a recent study aptly focused on TE lineage determination in bovine and found that the bovine *OCT4* promoter lacks the TCFAP2 binding sites that are responsible for early *OCT4* repression in the mouse TE [20]. However, the roles of collaborative factors such as *SOX2* and *NANOG* were not covered by that study. Our ISH results suggest that pluripotency may become restricted to the ICM quite earlier because of *NANOG* restriction to this lineage.

#### b) Expression profiles and spatial distribution of pluripotency proteins

Despite the presence of maternal *OCT4* transcripts, OCT4 protein was not detectable in oocytes or early embryos until it was first detected in the nuclei of early morula cells. In blastocysts, OCT4 was observed in cell nuclei of both the ICM and TE. These findings clearly corroborate earlier observations of OCT4 in the ICM and TE of bovine blastocysts [20], [22]. However, the only study in the literature to have addressed OCT4 detection in bovine during pre-EGA stages mentioned the detection of OCT4 in the oocyte and early embryos (before the 16-cell stage) [22]. Unfortunately, antibody specificity in that study was not verified using any method other than immunofluorescence. We performed Western Blot analysis which is in concordance with our immunofluorescence results. We suppose that technical differences, particularly with respect to antibody specificity, could have caused this contradiction with previous published results. The absence of OCT4 from oocytes and early embryos, however, presented an interesting paradox. In fact, OCT4 is found throughout early development in the mouse and is one of the maternal effect genes which may halt the embryonic development if it is disrupted [45]. Further, gene knockdown study in the mouse has emphasised its role during EGA [5]. Functional analyses are therefore necessary to precisely assign the role of OCT4 in early bovine embryos.

SOX2 was first observed in the nuclei of early bovine morulae. We assumed that the faint fluorescent signal observed before that stage is only unspecific background because it is exclusively cytoplasmic and is also observed in control experiments omitting the first antibody (data not shown). In mouse, maternal SOX2 has been shown to modulate gene reprogramming during EGA and is localized in the nucleus of the oocyte and 1-cell stage embryo [6]. According to our data this is not the case in bovine embryos. At a later stage, E7 blastocysts were characterized by the nuclear labelling of SOX2 in the ICM, while it was absent from the trophectoderm. Therefore, SOX2 seems to be a more pertinent candidate of pluripotent lineage specification than OCT4 in bovine.

In the pre-EGA phase, NANOG was not detectable using immunofluorescence until the 8-cell stage. Before that stage, the faint cytoplasmic immunofluorescent labelling was not different from that observed in control experiments omitting the first antibody (data not shown). All cells in the morula displayed nucleolar labelling. Blastocysts had similar nucleolar labelling in the TE, whereas in ICM cells both nucleoplasmic and nucleolar labelling were observed at E7, E8 and E9.

Such a nucleolar labelling for NANOG has not been described in mouse [46] and bovine embryos [47]. But a similar nucleoplasmic and nucleolar labelling has been described in the goat [48] and in early bovine blastocyst explants [47] using the same anti-NANOG antibody as us. To get further insight into this peculiar staining, we performed Western Blot analysis which revealed that oocyte and embryos up to the morula stage displayed a 28 kD band whereas embryos at E7 to E9 contained both 28 kD and 50 kD bands. The specificity of the 28 kD band was confirmed by pre-adsorption of the antibody solution with recombinant NANOG protein (data not shown). According to the literature [42] Nanog forms homodimers in order to exert its pluripotency function. We supposed that the 50 kD homodimeric form corresponds to the nucleoplasmic form in ICM cells, which are the pluripotent lineage. The 28 kD polypeptide would thus correspond to the nucleolar form which we hypothesized is recognized in immunofluorescence experiments only when concentrated in the nucleoli. It has been considered that the nucleolar protein would not be involved in pluripotency [47]. Moreover, the sequestration of proteins in the nucleoli has been proposed to be involved in the inactivation process that ultimately leads to ubiquitination and degradation of the transcription factor [49].

### 2. Expression profiles of SP/EP genes in early bovine embryos

In the present study, we analysed the expression profiles of some important genes involved in pluripotency signalling pathways or early patterning.

Our findings indicated that these transcripts were contributed maternally and were degraded during the MET process. The only exception is *MEIS1* which is up-regulated transiently at the 4-cell stage concomitant with bovine minor-EGA. These genes encode for factors involved in differentiation and patterning during development. In mice, for example, *Hesx1*is required for normal neuroectoderm formation [50], *Stat3* knockdown leads to embryonic death at day 6.5 [51], *Isl1* knockdown leads to developmental arrest and death at day 9.5 in mice [52] and *Nodal* is involved in patterning of early embryo during the mesoderm and endoderm formation [53]. Because the transcriptional activation of these genes does not occur immediately after EGA, it can therefore be assumed that they are down-regulated during early development and expressed later at appropriate time points during development. Interestingly in the early embryo the expression patterns of *STAT3*, *HESX1*, *MEIS1* and *ISL1* are globally similar (with maternal transcripts progressively degraded, and no expression at EGA) and seems opposite to the expression patterns of pluripotency master genes. This appears to differ from the situation described in mouse and human ES cells where *Meis1* and *Isl1* are down-regulated but *Stat3* and *Hesx1* are up-regulated because of Oct4, Sox2 and Nanog binding [12]. We first have to notice that maternal transcripts for these genes may have been expressed earlier during oogenesis when the expression of pluripotency factors has not been documented so far, then stably stored. Concerning the absence of embryonic transcription, these results point to the difference between ES cells and early embryo's genomes which are in different epigenetic states [54]. It is also worth mentioning that transcription is only poorly regulated in ES cells which are probably more prone to gene transcription than early embryonic genome [55]. Finally, we cannot exclude interspecies variations in the regulation of these genes by pluripotency factors.

### 3. Expression patterns of pluripotency genes and their SP/EP genes in cloned bovine embryos

The significance of gene knockdown strategies to analyse the functional importance of a particular gene is unquestionable, but they provide insufficient insights into the short or long term effects caused by small variations in the gene expression level. It is known that if an embryo acquires a proper gene expression pattern after EGA, embryonic development potential may be better, as embryonic death and senescence may ensue otherwise [56]. In this context, we hypothesised that the expression levels of pluripotency genes could be related to embryo development potential in bovine.

Intriguingly, the comparison of the three types of morulae showed that the morula with a poorer potential for development to term (Clone Morula B) expressed significantly higher levels of all of the genes analysed, including the pluripotency genes and their downstream targets (except for *MEIS1*). The systematic over-expression of nearly all the genes tested at the early morula stage was a unique observation which was not due to a significantly higher number of cells in the Clone Morula B. We thus assumed that Clone Morula B contained higher levels of a large proportion of its total messenger RNAs resulting from higher transcription rate. One may argue that higher transcript level at morula might have been due to malfunction of the degradation of maternally contributed factors such as *OCT4*, *SOX2*, *STAT3*, and *ISL1*. But an average three-fold increase in the level of transcripts in Clone Morula B compared to IVF morulae, seemed impossible to be due to degradation failure alone. This notion is supported by the higher levels of *NANOG* transcripts in Clone Morula B which has no maternal contribution. We thus conclude to a higher transcription rate of multiple genes including the pluripotency genes in Clone Morula B. More detailed analyses are necessary to ascertain the precise epigenetic reasons for this over-expression which at least for pluripotency genes was not due to a difference in their transcription rate in donor cells.

The functional consequences of gene overexpression on the embryonic development raised new questions. Indeed, normal embryonic development requires an appropriate formation of pluripotent epiblast and extraembryonic tissues. The over-expression of pluripotency genes may directly or indirectly affect these processes to cause embryonic death. This has been reported for a single gene over-expression [57], however, in Clone Morula B the three pluripotency genes (*OCT4*, *NANOG* and *SOX2*) were concomitantly over-expressed and their precise ratio remained the same as in IVF control embryos. This observation was interesting, because it has recently been proposed that *Oct4, Sox2* and *Nanog* could be lineage specifiers and a precarious balance between them would result in pluripotency [58]. If this supposition is true then Clone Morula B should be devoid of any detrimental effects on their early development and blastocyst formation. The higher levels of pluripotency genes could result in developmental failure of Clone Morula B at later stage when the balance between these genes becomes disturbed. This explanation is compatible with experimental data since Clone Morula A and B had similar rates of blastocyst formation; however, after embryo transfer Clone Morula B displayed morphological abnormalities at peri-implantation stages (Degrelle et al. in preparation) and rarely developed to birth. In addition, gene over-expression is not limited to pluripotency genes but concerns a large number of genes in Clone Morula B. We suppose that the over-expression of pluripotency factors could be responsible for a global higher transcription rate after EGA in bovine as evidenced at EGA in the mouse [5], [6].

In conclusion, the results of the present study provide valuable new insights into the gene dynamics in the particular context of maternal to embryonic transition (MET) and early lineage differentiation. Our findings affirm that firstly, the transcripts of *OCT4* and *SOX2* arise from both the maternal and embryonic genomes, while *NANOG* is synthesised by the embryo alone and none of the proteins of these genes is of maternal origin, except for a monomeric form of NANOG which is supposed not to be functional. Therefore, these genes probably do not play major roles in bovine EGA. Secondly, bovine pluripotent lineage specification segues progressively in line with development. The transcript and protein localisation experiments show an earlier ICM specific compartmentalization of both SOX2 and NANOG compared to OCT4, thus these two genes represent interesting candidates for pluripotent lineage specification and require functional analyses. In addition, cloned embryos proved to be a complementary “real-life” alternative for the classic functional studies, which report correlation between the induced up or down-regulation of a single gene in the embryos and their developmental potential [57]. Our findings support the notion that the expression levels of pluripotency genes may presage the long term developmental potential of bovine embryos.
